# Early Life Origins of All-Cause and Cause-Specific Disability Pension: Findings from the Helsinki Birth Cohort Study

**DOI:** 10.1371/journal.pone.0122134

**Published:** 2015-04-07

**Authors:** Mikaela B. von Bondorff, Timo Törmäkangas, Minna Salonen, Monika E. von Bonsdorff, Clive Osmond, Eero Kajantie, Johan G. Eriksson

**Affiliations:** 1 Gerontology Research Center and Department of Health Sciences, University of Jyvaskyla, Jyväskylä, Finland; 2 Division of Welfare and Health Promotion, Department of Chronic Disease Prevention, Diabetes Prevention Unit, National Institute for Health and Welfare, Helsinki, Finland; 3 Folkhälsan Research Centre, Helsinki, Finland; 4 MRC Lifecourse Epidemiology Unit, University of Southampton, Southampton, United Kingdom; 5 Hospital for Children and Adolescents, Helsinki University Central Hospital and University of Helsinki, Helsinki, Finland; 6 Department of Obstetrics and Gynaecology, Oulu University Hospital and University of Oulu, Oulu, Finland; 7 Department of General Practice and Primary Health Care, University of Helsinki, Helsinki, Finland; 8 Unit of General Practice, Helsinki University Central Hospital, Helsinki, Finland; 9 Vasa Central Hospital, Vasa, Finland; Hunter College, UNITED STATES

## Abstract

**Background:**

There is some evidence linking sub-optimal prenatal development to an increased risk of disability pension (DP). Our aim was to investigate whether body size at birth was associated with transitioning into all-cause and cause-specific DP during the adult work career.

**Methods:**

10 682 people born in 1934–44 belonging to the Helsinki Birth Cohort Study had data on birth weight extracted from birth records, and on time, type and reason of retirement between 1971 and 2011 extracted from the Finnish Centre for Pensions.

**Results:**

Altogether 21.3% transitioned into DP during the 40-year follow-up, mainly due to mental disorders, musculoskeletal disorders and cardiovascular disease. Average age of transitioning into DP was 51.3 (SD 8.4) for men and 52.2 (SD 7.6) for women. Cohort members who did not transition into DP retired 10 years later on average. Among men, higher birth weight was associated with a lower hazard of transitioning into DP, adjusted hazard ratio (HR) being 0.94 (95% confidence interval [CI] 0.88–0.99 for 1 SD increase in birth weight). For DP due to mental disorders the adjusted HR was 0.90, 95% CI 0.81, 0.99. A similar but non-significant trend was found for DP due to cardiovascular disease. Among women there were no associations between body size at birth and all-cause DP (p for interaction gender*birth weight on DP p = 0.007).

**Conclusions:**

Among men disability pension, particularly due to mental disorders, may have its origins in prenatal development. Given that those who retire due to mental health problems are relatively young, the loss to the workforce is substantial.

## Introduction

Disability pension (DP), considered as a permanent exit from the workforce due to a medical cause, has been increasing in Europe over the past decades and it has significant economic implications at both the individual and societal level [1]. In 2011, of all Finnish residents retiring on a pension based on their own work history, 32.1% transitioned into DP at an average age of 52.1 years [2]. Due to increasing life expectancy and current low birth rates [3], the pressure to raise the retirement age is growing. Thus better knowledge of the determinants of DP could yield substantial benefits for individuals and the society in prolonging working careers.

Research on underlying causes of transitioning into DP has mostly focused on socioeconomic status in childhood and adulthood, related health conditions [4,5] and on features of the work [6]. According to the critical period model, which is one of the original life course epidemiology models [7] suboptimal prenatal conditions may cause long-lasting changes in the developing organ structures and functioning of biological systems placing an individual at risk of disease in adulthood [8]. So far the contribution of developmental origins of health and disease to the risk of transition into DP has been scarcely investigated, although the link is theoretically plausible.

We have previously shown in the Helsinki Birth Cohort that small body size at birth, a consequence of an adverse prenatal environment and suboptimal growth, is associated with a higher risk of coronary heart disease [9], mental disorders such as schizophrenia [10] and physical functioning [11] in adulthood. But, to the best of our knowledge, whether this established association is also reflected in transitioning into DP due to mental disorders, musculoskeletal disorders or cardiovascular disease has not been previously investigated. We were, however, able to identify two earlier studies on prenatal development and the risk of all-cause DP. In a large Norwegian cohort [12] the risk of early DP was higher for those with standardized birth weight below the mean, particularly among men with lower educational attainment. Echoing these findings, a recent Swedish study [13] found that sparing of key organs at the expense of fat formation and muscle development as a result of growth retardation during the 3^rd^ trimester led to babies being born asymmetrically small for gestational age (A-SGA), and these babies had increased risk of DP. Based on these earlier findings we hypothesized that smaller body size at birth was associated with a higher risk of subsequent disability pension among men and women. Using data from the Helsinki Birth Cohort Study the aim of the present study was to investigate if birth weight was associated with transitioning into all-cause and cause-specific disability pension during the adult work career and whether childhood and adulthood socioeconomic status explained the potential association.

## Materials and Methods

### Study population

The Helsinki Birth Cohort Study comprises 13 345 individuals born in Helsinki, Finland at Helsinki University Central Hospital or Helsinki City Maternity Hospital between 1934 and 1944 and who had data on birth anthropometry extracted from birth records [9,14]. Of the original cohort members, 11 378 had retired by the end of follow-up in December 2011 and had data available on date and type of pension and primary diagnosis for disability pension provided by the Finnish Centre for Pensions. From the original cohort, 1800 persons had either migrated or died without a retirement decision. We excluded 167 persons who had no data available on retirement, migration or death. Of these 11 378 cohort members, 10 682 (93.9%) had complete data available on the main variables and covariates used in these analyses. Birth weight and length did not differ among those 10 682 in the present analyses and the 2663 with missing data (*t*-test p>0.143). The study was approved by the Ethics Committee of Epidemiology and Public Health of the Hospital District of Helsinki and Uusimaa and that of the National Public Health Institute, Helsinki. Written consent was not given by the cohort members, however, the data were anonymized prior to analysis.

### Retirement data

All Finnish residents are covered by the pension system. Before reaching the statutory retirement age employees can apply for disability pension if, due to a medically confirmed illness, they are unable to continue working even after periods of rehabilitation, re-education or assistance [2]. Using the unique personal identification number, we were able to retrieve pension data from January 1971 to December 2011, thus we have data on retirement from an average age of 30 years (range 26 to 37) to 71 years (range 67 to 78). During the follow-up, the statutory retirement age for e.g. municipal employees was 63 years [15], indicating that we were likely to capture the retirement of all cohort members. For these analyses, we coded pension type as (all-cause) disability pension vs. non-disability pension, which included all other types of pension (old-age, early old age, part-time, unemployment and early individual retirement). Further, we coded the main causes of disability pension according to the International Classification of Diseases (ICD) 9^th^ and 10^th^ versions into the following groups: mental disorders (ICD 9 codes 290–319 and ICD 10 codes F00-F99), diseases of the musculoskeletal system (710–739 and M00-M99); cardiovascular diseases (CVD) (400–459 and I00-I99); diseases of the nervous system (320–359 and G00-G99); and all other diagnoses.

### Birth, childhood and adulthood measures

Birth date, weight, length and birth order of the newborns were retrieved from the hospital birth records described in detail previously [9,16,17]. Body mass index (BMI) was calculated as weight (kg) divided by height (m) squared. Birth order, ranging from firstborn to fifteenth born, was coded as firstborn versus second or higher. Childhood socioeconomic status was ascertained based on father’s highest occupation status extracted from birth, child welfare and school healthcare records and coded as upper middle class, lower middle class and manual workers based on the original social classification system issued by Statistics of Finland [18]. Register data from Statistics Finland, obtained at 5-year intervals between 1970 and 2000 were used to indicate adult socioeconomic status. Highest educational attainment was coded as basic/primary or less, upper secondary, lower tertiary and upper tertiary. Highest occupational status before retirement was coded as upper middle class, lower middle class, self-employed and manual workers [18].

### Statistical analyses

Birth weight was standardized into a Z-score indicating the number of standard deviations by which an observation differs from the mean of the entire study cohort. To test for potential gender differences we first analyzed the interaction ‘gender*birth weight’ on DP in a Cox regression model and because the term was statistically significant (p = 0.007), subsequent analyses were stratified by gender. Nelson-Aalen cumulative hazards of retirement were calculated for the 40-year follow-up time in groups according to non-DP and DP due to mental disorders, musculoskeletal disorders, cardiovascular diseases and all other diagnoses.

Using Cox proportional hazards models we estimated the hazards and their 95% confidence intervals for transitioning into DP during the 40-year follow-up for birth weight. Follow-up time was calculated as the number of days between date of birth and date of retirement. In these models non-disability pensioners were treated as censored cases while each disability pension category in turn was treated as an event. Separate analyses were conducted for the main disability pension diagnoses. The proportional hazards assumption was assessed using a test based on scaled Schoenfeld residuals to identify variables for which associations varied over time. Diagnostic investigations indicated that only the effect of birth weight on retirement due to mental disorders among women was not proportional over time. In the Cox models, after the crude model, we first adjusted for birth order and socioeconomic status in childhood and then for highest educational attainment and highest occupational class in adulthood. Cox models were stratified on year of birth to control for potential cohort effects. Modeling was performed with IBM SPSS version 22.0 except for the Nelson-Aalen Cumulative Hazard computation and Schoenfeld residual checks which were performed in the R statistical programming environment (version 3.0.3) using the survival-package (version 2.37–7).

## Results

Altogether 21.3% (23.5% men and 18.9% women) of the study members transitioned into DP during the 40-year follow-up period. The average age of transitioning into DP was 51.3 years (SD 8.4) for men and 52.2 years (SD 7.6) for women whereas those who transitioned into non-disability pension worked on average 10 years longer and retired at an average age of 61 years (see Table 1). Birth weight was lower for men who transitioned into DP compared to those who transitioned into non-DP (t-test p = 0.012). Among women there were no such associations. Those who transitioned into DP were less frequently firstborns and more often had fathers working in manual professions. There were large differences in terms of adult socioeconomic status showing that those who transitioned into DP had at most secondary education more frequently and were manual workers more frequently compared to non-DP pensioners (χ^2^ test p<0.001).

**Table 1:** Characteristics of men and women in the Helsinki Birth Cohort Study for occurrence of disability pension and non-disability pension.

	**Men**				**Women**			
	All	Disability pension	Non-disability	p	All	Disability pension	Non-disability	p
	n = 5497	n = 1290	pension n = 4207		n = 5185	n = 981	pension n = 4204	
Birth anthropometrics, mean, SD								
Weight, kg	3.47, 0.49	3.44, 0.51	3.48, 0.48	0.012	3.35, 0.45	3.36, 0.46	3.34, 0.45	0.188
Length, cm	50.58, 1.96	50.50, 2.07	50.61, 1.92	0.075	49.93, 1.78	49.93, 1.75	49.93, 1.78	0.98
Body mass index, weight/(height)^2^	13.50, 1.24	13.43, 1.28	13.53, 1.23	0.011	13.37, 1,23	13.44, 1.26	13.36, 1.22	0.056
Birth order, %				0.004				0.047
Firstborn	49.0	45.6	50.1		47.3	44.4	48.0	
Second or higher	51.0	54.4	49.9		52.7	55.6	52.0	
Father’s occupational status, %				<0.001				<0.001
Upper middle	18.8	13.4	20.4		16.7	12.9	17.6	
Lower middle	24.2	20.9	25.3		24.4	21.6	25.0	
Manual worker	57.0	65.7	54.3		58.9	65.5	57.4	
Highest educational attainment, %				<0.001				<0.001
Upper tertiary	13.6	5.1	16.2		8.5	3.9	9.5	
Lower tertiary	23.9	15.4	26.5		23.1	16.4	24.7	
Upper secondary	26.0	29.7	24.9		23.9	25.9	23.5	
Basic or less	36.5	49.8	32.4		44.5	53.8	42.3	
Adult occupational status, %				<0.001				<0.001
Upper middle	48.2	27.6	54.5		41.2	26.8	44.6	
Lower middle	24.9	26.7	24.3		49.8	57.1	48.0	
Self-employed	5.9	8.8	5.0		2.7	3.4	2.6	
Manual worker	21.0	36.9	16.2		6.3	12.7	4.8	
Age at transition to pension, years								
mean SD	58.9, 6.6	51.3, 8.4	61.2, 3.4	<0.001	59.6, 5.5	52.2, 7.6	61.3, 2.8	<0.001
Cause of disability pension, %								
Mental disorders		29.1				35.9		
Musculoskeletal disorders		18.1				27.0		
Cardiovascular disease		17.9				7.3		
Diseases of the nervous system		6.4				7.7		
All other diagnoses		28.5				22.1		

**Fig 1:**
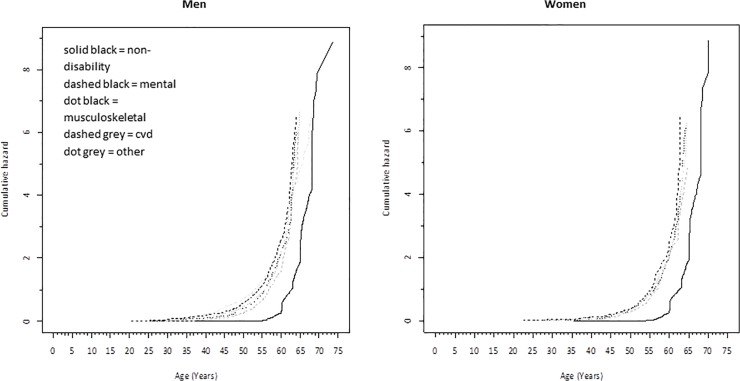
Nelson-Aalen cumulative hazards of retirement in groups according to non-DP and DP due to the main diagnostic groups.

The two leading causes of DP were mental disorders (main diagnoses were depressive episodes, neurotic disorders, mood disorders and schizophrenia) and musculoskeletal disorders (main diagnoses were intervertebral disc disorders, osteoarthritis and arthrosis of knee) and the other main causes were CVD and diseases of the nervous system. Nelson-Aalen Cumulative Hazards were calculated for non-DP and DP due to the main diagnoses (mental disorders, musculoskeletal disorders, CVD and all other diagnoses) for the 40-year follow-up period (Fig 1). The cumulative hazards of retirement were elevated early in the follow-up for main DP groups compared to the non-DP group. The cumulative hazard started to rise after age 20 years and was consistently higher throughout the working career for those who transitioned into DP due to mental disorders. Differences in characteristics of the study members across the main causes of DP are presented in S1 Table and S2 Table for men and women, respectively. From the main groups of diagnoses the ones who retired due to mental disorders were the youngest to retire compared to the rest of the cohort. Over 70% of the men and women who transitioned into DP because of musculoskeletal disorders had a father who worked in a manual profession and they also worked most frequently in a manual profession themselves before transitioning into DP.

Cox regression models indicated that higher birth weight among men was associated with a lower hazard of all-cause DP in the follow-up, HR 0.93 (95% CI 0.88–0.98 for 1 SD increase in birth weight) (Table 2). The association was not explained by birth order or socioeconomic status in childhood or adulthood nor did we find any statistically significant interactions between birth weight and socioeconomic status in childhood or adulthood on DP (p>0.25). Among women birth weight was not associated with the hazard of all-cause DP. Next, we investigated the hazards for DP due to the main diagnoses (Table 3). Cox regression models showed that higher birth weight among men was associated with decreased risk of DP due to mental disorders, the adjusted HR being 0.90 (95% CI 0.81, 0.99). A similar but non-significant trend was found for birth weight and DP due to CVD among men. Among women there were no associations between birth weight and the main diagnoses of DP. However, diagnostic tests and plots on scaled Schoenfeld residuals suggested that the proportional hazards assumption of the association between birth weight and retirement due to mental disorders among women changed significantly over time (p<0.001). These plots indicated that, up to age 55 years, higher birth weight was not associated with DP due to mental disorders, adjusted HR 0.99 (95% CI 0.85–1.16). After the age of 55 years the association was positive, adjusted HR 1.23 (95% CI 1.03–1.47).

**Table 2:** Hazard ratios (HR) and 95% CIs for disability vs. non-disability pension in adulthood according to birth weight in the Helsinki Birth Cohort Study.

	**Men**			**Women**		
	Model 1	Model 2	Model 3	Model 1	Model 2	Model 3
	HR (95% CI)	HR (95% CI)	HR (95% CI)	HR (95% CI)	HR (95% CI)	HR (95% CI)
Birth weight, z-score	0.93 (0.88–0.98)	0.91 (0.86–0.97)	0.94 (0.88–0.99)	1.04 (0.97–1.11)	1.03 (0.97–1.10)	1.05 (0.98–1.12)
Birth order						
Firstborn		1.00	1.00		1.00	1.00
Second or higher		1.19 (1.06–1.33)	1.08 (0.96–1.21)		1.10 (0.96–1.25)	1.03 (0.91–1.18)
Father’s occupational status						
Upper middle		1.00	1.00		1.00	1.00
Lower middle		1.24 (1.02–1.50)	1.00 (0.83–1.21)		1.17 (0.94–1.46)	1.00 (0.80–1.25)
Manual worker		1.71 (1.45–2.01)	1.09 (0.92–1.29)		1.52 (1.25–1.84)	1.08 (0.89–1.32)
Educational attainment						
Upper tertiary			1.00			1.00
Lower tertiary			1.57 (1.19–2.09)			1.40 (0.98–2.01)
Upper secondary			1.83 (1.37–2.45)			1.75 (1.22–2.51)
Basic or less			2.16 (1.62–2.87)			1.88 (1.32–2.68)
Adult occupational status						
Upper middle			1.00			1.00
Lower middle			1.62 (1.37–1.91)			1.61 (1.37–1.89)
Self-employed			2.06 (1.64–2.60)			1.37 (1.20–2.52)
Manual worker			2.89 (2.45–3.42)			3.18 (2.52–4.00)

Model 1 crude, Model 2 adjusted for birth order and father’s occupational status, Model 3 adjusted for Model 2 + educational attainment and adult occupational status.

**Table 3:** Hazard ratios (HR) and 95% CIs for disability pension (DP) due to mental disorders, musculoskeletal disorders and cardiovascular disease compared to non-disability pension according to birth weight in the Helsinki Birth Cohort Study.

	**DP due to mental disorder n = 375 vs.**		**DP due to musculoskeletal disorder n = 233**		**DP due to CVD n = 231 vs.**	
	**non-DP n = 4207**		**vs. non-DP n = 4207**		**non-DP n = 4207**	
	Model 1	Model 2	Model 1	Model 2	Model 1	Model 2
**Men**	HR (95% CI)	HR (95% CI)	HR (95% CI)	HR (95% CI)	HR (95% CI)	HR (95% CI)
Birth weight, z-score	0.88 (0.80–0.98)	0.90 (0.81–0.99)	1.01 (0.89–1.15)	1.02 (0.89–1.17)	0.90 (0.79–1.02)	0.88 (0.78–1.02)
Birth order						
Firstborn		1.00		1.00		1.00
Second or higher		0.94 (0.76–1.16)		1.13 (0.86–1.47)		1.25 (0.96–1.64)
Father’s occupational status						
Upper middle		1.00		1.00		1.00
Lower middle		0.82 (0.59–1.14)		1.76 (1.00–3.11)		1.22 (0.77–1.95)
Manual worker		0.88 (0.66–1.17)		1.94 (1.15–3.27)		1.33 (0.87–2.02)
Educational attainment						
Upper tertiary		1.00		1.00		1.00
Lower tertiary		1.57 (1.19–2.09)		2.96 (1.02–8.59)		2.74 (1.33–5.65)
Upper secondary		1.83 (1.37–2.45)		5.29 (1.85–15.18)		2.29 (1.08–4.89)
Basic or less		2.16 (1.62–2.87)		4.91 (1.72–14.04)		3.72 (1.79–7.75)
Adult occupational status						
Upper middle		1.00		1.00		1.00
Lower middle		1.27 (0.82–1.95)		1.64 (1.08–2.48)		1.45 (1.01–2.08)
Self-employed		1.22 (0.76–1.94)		3.25 (1.97–5.37)		1.93 (1.14–3.25)
Manual worker		1.49 (0.95–2.35)		3.97 (2.67–5.91)		2.16 (1.47–3.18)
**Women**	**DP due to mental disorder n = 352 vs.**		**DP due to musculoskeletal disorder n = 265**		**DP due to CVD n = 71 vs.**	
	**non-DP n = 4204**		**vs. non-DP n = 4204**		**non-DP n = 4204**	
Birth weight, z-score	0.95 (0.86–1.06)	0.96 (0.86–1.07)	1.08 (0.95–1.22)	1.08 (0.95–1.22)	0.89 (0.70–1.13)	0.93 (0.73–1.19)
Birth order						
Firstborn		1.00		1.00		1.00
Second or higher		1.05 (0.84–1.30)		1.17 (0.91–1.50)		0.83 (0.51–1.34)
Father’s occupational status						
Upper middle		1.00		1.00		1.00
Lower middle		0.91 (0.65–1.27)		1.24 (0.76–2.00)		0.70 (0.31–1.57)
Manual worker		0.85 (0.63–1.16)		1.39 (0.90–2.15)		0.95 (0.47–1.90)
Educational attainment						
Upper tertiary		1.00		1.00		1.00
Lower tertiary		1.14 (0.71–1.84)		2.86 (0.86–9.51)		2.41 (0.54–10.80)
Upper secondary		0.99 (0.60–1.64)		6.07 (1.87–19.71)		2.76 (0.60–12.61)
Basic or less		1.20 (0.74–1.84)		6.20 (1.92–20.07)		2.72 (0.60–12.27)
Adult occupational status						
Upper middle		1.00		1.00		1.00
Lower middle		1.65 (1.27–2.15)		1.75 (1.26–2.43)		1.67 (0.92–3.03)
Self-employed		1.18 (0.54–2.56)		1.78 (0.87–3.66)		1.30 (0.29–5.75)
Manual worker		2.94 (1.94–4.44)		4.06 (2.65–6.23)		3.47 (1.46–8.20)

Model 1 crude, Model 2 adjusted for birth order and father’s occupational status, Model 3 adjusted for Model 2 + educational attainment and adult occupational status.

We conducted sensitivity analyses by first using ponderal index (kg/m^3^) instead of birth weight in the Cox regression models and found the associations to be similar. Then, we further adjusted the models for gestational age, mother’s BMI and age, but they did not attenuate the associations between birth weight and DP. We added the quadratic and cubic terms of birth weight in the Cox regression models but because these terms were not significant we did not include them in the final models. Finally, we included the 1800 persons in the HBCS who had died or migrated and did not retire in the Cox regression models as censored cases, but this did not change the results.

## Discussion

In this large birth cohort with 40 years of follow-up from 1971–2011 higher birth weight among men was associated with decreased risk of transitioning into disability pension. The cohort members who retired due to mental disorders were the youngest to retire at an average age of 50 whereas those who transitioned into non-DP worked 10 years longer. For the first time we were able to investigate the contribution of prenatal factors in connection with the main causes of disability pension. Higher birth weight among men was associated with a lower risk of DP primarily due to mental disorders. Allowing for childhood and adulthood socioeconomic status did not attenuate the association suggesting that birth weight, an indicator of the prenatal environment of the fetus, was independently linked to the subsequent risk of DP.

Our findings are in line with the two Nordic studies that have previously investigated the link between intrauterine development and DP of any cause. In the first study by Gravseth et al. [12], a standardized birth weight below mean increased the risk of early DP among individuals born 1967–1976. They found the association to be particularly strong for those with lower educational attainment. In the other recent study by Helgertz et al. [13], being born asymmetrically small for gestational age was linked to an increased risk of DP. In the first study [12] the associations were stronger for men than for women, which supports our findings of a gender difference. The second study [13] did not stratify by gender. The significant findings of the present study for men but not women may be due to faster growth of the male fetus in utero rendering them more vulnerable to the effects of undernutrition [19,20], to the observed gender differences to stress responses for those with low birth weight [21] or to the differences in the level of job strain during the work career. For example, women were more often homemakers at some point and thus had more spells of not being employed and may have been less exposed to job strain during that time. Job strain has been linked with a higher risk of DP [6], which might have, along with non-optimal prenatal growth, contributed to the subsequent risk of DP among men.

The link between prenatal environment, of which birth weight is a marker, and DP is plausible. There is evidence from cohorts around the world supporting the ‘developmental origins of health and disease’ [8] model in showing that small body size at birth increased the risk of chronic disease such as CVD and type 2 diabetes in adulthood [22,23]. We did not find an association between birth size and DP due to CVD, but the trend was in line with earlier findings also reported in this cohort [9]. This finding suggests that the disability pension process is complex and not solely disease-driven, but that there may be other non-medical factors such as the mode of disability compensation system [24] or high job strain [6], affecting the disability retirement process.

A higher prevalence of mental health disorders such as depression and schizophrenia have been linked to suboptimal prenatal development i.e. lower birth weight and shorter stature [10,25,26] although contrasting findings have been reported [27]. We found that smaller body size at birth was linked with an increased risk of DP due to mental disorders in men, which is in line with findings from other studies [26,28]. There may be several mechanisms through which intrauterine development might increase susceptibility to mental disorders in adulthood causing early exit from the workforce. First, abnormalities in the maturation of the brain may stem from fetal development [29]. In neurodevelopmental studies magnetic resonance imaging confirmed that children born small for gestational age had smaller brain volume in adolescence [30] and that lower ponderal index among term born babies was associated with accelerated brain atrophy in old age [31]. A stimulus or insult during a sensitive time-window in development can have permanent effects on neurodevelopment which has been shown to contribute to personality dimensions such as neuroticism [32] which themselves have been further linked with a higher prevalence of depressive symptoms [33]. Secondly, neuroendocrine systems, particularly those regulating stress responses such as the hypothalamic-pituitary-adrenal (HPA) axis, have been shown to be affected by suboptimal prenatal development [34,35]. An earlier study found that low birth weight among boys but not girls was related to a higher salivary cortisol response, an indicator of HPA axis reactivity [21,36] which might predispose these individuals to a higher incidence of mental disorders [37]. These differences between boys and girls might be one explanation for our non-significant finding among women.

The strengths of our study include the well-characterized sample and information on birth size collected from birth records. Register data on pensions were available for practically all original cohort members who had not migrated or died before retiring, thus minimizing loss to follow-up. We were also able to use register data on adult socioeconomic status. Some limitations of the study should be recognized. Birth weight is a crude measure of the intrauterine environment. However, it has been extensively used in the literature as a marker of prenatal development. The relationship between birth weight and DP was independent of length of gestation, suggesting that it can be attributed to slow fetal growth rather than preterm birth. We were however able to control for a limited number of early exposures and thus there might have been other confounders which potentially explain the present findings. The individuals in this study were born in the two public hospitals of the city of Helsinki, they had attended voluntary child-welfare clinics that were free of charge and the majority went to school in Helsinki. Thus, the participants may not represent the entire population living in Finland. However, at birth, childhood social class as indicated by fathers highest occupational status did not differ from that of the population living in the city of Helsinki at that time [38]. In this historical cohort, most individuals were born or grew up during the Second World War, a time during which families might have suffered from food shortages in Finland. This needs to be considered when generalizing these results into contemporary cohorts. In addition, we did not have enough statistical power to investigate the association between birth size and specific diseases or health conditions such as depression.

In conclusion, in this large birth cohort and in 40-years of follow-up we found that higher birth weight was associated with a lower risk of disability pension among men in diverse occupations. The association was particularly strong for men who retired due to mental disorders. This piece of information is important, considering that the loss in workforce due to DP is considerable, particularly among those who transition into DP due to mental disorders, which often takes place at an earlier age than other disabilities. More information is needed on the contribution of early life factors to disability pension due to specific diseases such as depression.

## Supporting Information

S1 Table Characteristics of men in the Helsinki Birth Cohort Study according to non-disability pension and disability pension due to main diagnoses, n = 5497. (DOCX)

S2 Table Characteristics of women in the Helsinki Birth Cohort Study according to non-disability pension and disability pension due to main diagnoses, n = 5185. (DOCX)

## References

[1] OECD. Sickness, disability and work: Breaking the barriers A synthesis of findings across OECD countries. Paris: OECD Publishing; 2010.

[2] Official Statistics of Finland. Pensioners and insured in Finland 2011. Helsinki: Finnish Center for Pensioners; 2013.

[3] OECD. Pensions at a Glance 2011: Retirement income systems in OECD and G20 countries Paris: OECD Publishing; 2011.

[4] Krokstad S, Johnsen R, Westin S. Social determinants of disability pension: a 10-year follow-up of 62 000 people in a Norwegian county population. Int J Epidemiol 2002;31:1183–91. 12540720 10.1093/ije/31.6.1183

[5] Harkonmäki K, Korkeila K, Vahtera J, Kivimäki M, Suominen S, Sillanmäki L, et al Childhood adversities as a predictor of disability retirement. J Epidemiol Community Health 2007;61:479–84. 17496255 10.1136/jech.2006.052670 PMC2465717

[6] Laine S, Gimeno D, Virtanen M, Oksanen T, Vahtera J, Elovainio M, et al Job strain as a predictor of disability pension: the Finnish Public Sector Study. J Epidemiol Community Health 2009;63:24–30. 10.1136/jech.2007.071407 18768568

[7] Kuh D, Ben-Shlomo Y, Lynch J, Hallqvist J, Power C. Life course epidemiology. J Epidemiol Community Health 2003;57:778–83. 14573579 10.1136/jech.57.10.778 PMC1732305

[8] Barker DJ. The developmental origins of adult disease. J Am Coll Nutr 2004;23:588S–95S. 15640511 10.1080/07315724.2004.10719428

[9] Eriksson JG, Forsen T, Tuomilehto J, Winter PD, Osmond C, Barker DJ. Catch-up growth in childhood and death from coronary heart disease: longitudinal study. BMJ 1999;318:427–31. 9974455 10.1136/bmj.318.7181.427 PMC27731

[10] Wahlbeck K, Forsen T, Osmond C, Barker DJ, Eriksson JG. Association of schizophrenia with low maternal body mass index, small size at birth, and thinness during childhood. Arch Gen Psychiatry 2001;58:48–52. 11146757 10.1001/archpsyc.58.1.48

[11] von Bonsdorff MB, Rantanen T, Sipilä S, Salonen MK, Kajantie E, Osmond C, et al Birth size and childhood growth as determinants of physical functioning in older age: the Helsinki Birth Cohort Study. Am J Epidemiol 2011;174:1336–44. 10.1093/aje/kwr270 22071586

[12] Gravseth HM, Bjerkedal T, Irgens LM, Aalen OO, Selmer R, Kristensen P. Life course determinants for early disability pension: a follow-up of Norwegian men and women born 1967–1976. Eur J Epidemiol 2007;22:533–43. 17530421 10.1007/s10654-007-9139-9

[13] Helgertz J, Vagero D. Small for gestational age and adulthood risk of disability pension: The contribution of childhood and adulthood conditions. Soc Sci Med 2014;119:249–57. 10.1016/j.socscimed.2013.11.052 24423878

[14] Forsen T, Eriksson JG, Tuomilehto J, Teramo K, Osmond C, Barker DJ. Mother's weight in pregnancy and coronary heart disease in a cohort of Finnish men: follow up study. BMJ 1997;315:837–40. 9353502 10.1136/bmj.315.7112.837 PMC2127571

[15] Ilmarinen J, Suurnäkki T, Nygard CH, Landau K. Classification of municipal occupations. Scand J Work Environ Health 1991;17 Suppl 1:12–29. 1792524

[16] Barker DJ, Osmond C, Forsen TJ, Kajantie E, Eriksson JG. Trajectories of growth among children who have coronary events as adults. N Engl J Med 2005;353:1802–9. 16251536 10.1056/NEJMoa044160

[17] Osmond C, Kajantie E, Forsen TJ, Eriksson JG, Barker DJ. Infant growth and stroke in adult life: the Helsinki birth cohort study. Stroke 2007;38:264–70. 17218608 10.1161/01.STR.0000254471.72186.03

[18] Central Statistical Office of Finland. Classification of socioeconomic groups: handbooks 17 Helsinki, Finland: Central Statistical Office of Finland; 1989.

[19] Barker DJ. Mothers, babies and health in later life London: Churchill Livingstone; 1998.

[20] Lampl M, Gotsch F, Kusanovic JP, Gomez R, Nien JK, Fronqillo EA, et al Sex differences in fetal growth responses to maternal height and weight. Am J Hum Biol 2010;22:431–43. 10.1002/ajhb.21014 19950190 PMC3437780

[21] Jones A, Godfrey KM, Wood P, Osmond C, Goulden P, Phillips DI. Fetal growth and the adrenocortical response to psychological stress. J Clin Endocrinol Metab 2006;91:1868–71. 16464950 10.1210/jc.2005-2077

[22] Whincup PH, Kaye SJ, Owen CG, Huxley R, Cook DG, Anazawa S, et al Birth weight and risk of type 2 diabetes: a systematic review. JAMA 2008;300:2886–97. 10.1001/jama.2008.886 19109117

[23] Huxley R, Owen CG, Whincup PH, Cook DG, Rich-Edwards J, Smith GD, et al Is birth weight a risk factor for ischemic heart disease in later life? Am J Clin Nutr 2007;85:1244–50. 17490959 10.1093/ajcn/85.5.1244

[24] OECD. Transforming disability into ability: Policies to promote work and income security for disabled people Paris: OECD publication service; 2003.

[25] Räikkönen K, Pesonen AK, Kajantie E, Heinonen K, Forsen T, Phillips DI, et al Length of gestation and depressive symptoms at age 60 years. Br J Psychiatry 2007;190:469–74. 17541105 10.1192/bjp.bp.106.022145

[26] Thompson C, Syddall H, Rodin I, Osmond C, Barker DJ. Birth weight and the risk of depressive disorder in late life. Br J Psychiatry 2001;179:450–5. 11689404 10.1192/bjp.179.5.450

[27] Osler M, Nordentoft M, Andersen AM. Birth dimensions and risk of depression in adulthood: cohort study of Danish men born in 1953. Br J Psychiatry 2005;186:400–3. 15863744 10.1192/bjp.186.5.400

[28] Hultman CM, Sparen P, Takei N, Murray RM, Cnattingius S. Prenatal and perinatal risk factors for schizophrenia, affective psychosis, and reactive psychosis of early onset: case-control study. BMJ 1999;318:421–6. 9974454 10.1136/bmj.318.7181.421 PMC27730

[29] Rees S, Harding R. Brain development during fetal life: influences of the intra-uterine environment. Neurosci Lett 2004;361:111–4. 15135906 10.1016/j.neulet.2004.02.002

[30] Martinussen M, Fischl B, Larsson HB, Skranes J, Kulseng S, Vangberg TR, et al Cerebral cortex thickness in 15-year-old adolescents with low birth weight measured by an automated MRI-based method. Brain 2005;128:2588–96. 16123146 10.1093/brain/awh610

[31] Muller M, Sigurdsson S, Kjartansson O, Jonsson PV, Garcia M, von Bonsdorff MB, et al Birth Size and Brain Function 75 Years Later. Pediatrics 2014;134:1–10. 25180277 10.1542/peds.2014-1108 PMC4179101

[32] Lahti M, Räikkönen K, Lemola S, Lahti J, Heinonen K, Kajantie E, et al Trajectories of physical growth and personality dimensions of the Five-Factor Model. J Pers Soc Psychol 2013;105:154–69. 10.1037/a0032300 23713700

[33] Steunenberg B, Beekman AT, Deeg DJ, Kerkhof AJ. Personality predicts recurrence of late-life depression. J Affect Disord 2010;123:164–72. 10.1016/j.jad.2009.08.002 19758704

[34] Kajantie E, Feldt K, Räikkönen K, Phillips DI, Osmond C, Heinonen K, et al Body size at birth predicts hypothalamic-pituitary-adrenal axis response to psychosocial stress at age 60 to 70 years. J Clin Endocrinol Metab 2007;92:4094–100. 17848405 10.1210/jc.2007-1539

[35] Phillips DI, Jones A. Fetal programming of autonomic and HPA function: do people who were small babies have enhanced stress responses? J Physiol 2006;572:45–50. 16455684 10.1113/jphysiol.2005.104695 PMC1779633

[36] Kajantie E, Räikkönen K. Early life predictors of the physiological stress response later in life. Neurosci Biobehav Rev 2010;35:23–32. 19931557 10.1016/j.neubiorev.2009.11.013

[37] Gunnar M, Quevedo K. The neurobiology of stress and development. Annu Rev Psychol 2007;58:145–73. 16903808 10.1146/annurev.psych.58.110405.085605

[38] Siipi J. Pääkaupunkiyhteiskunta ja sen sosiaalipolitiikka In: Rosen R, Hornborg E, Jutikkala E, Waris H, Castren M, editors. Helsingin kaupungin historia, 5. osa, 1. nide, Ajanjakso 1918–1945 Helsingissä. Helsinki: City of Helsinki; 1962 p. 137.

