# Supplementary material for: Early Life Origins of All-Cause and Cause-Specific Disability Pension: Findings from the Helsinki Birth Cohort Study
Source: PLoS One. 2015 Apr 7;10(4):e0122134. doi: 10.1371/journal.pone.0122134 (PMC4388659; doi:10.1371/journal.pone.0122134)
Supplement: S2 Table — (DOCX) [file pone.0122134.s002.docx]

Table S2 Characteristics of women in the Helsinki Birth Cohort Study according to non-disability pension and disability pension due to main diagnoses, n=5185

|  | Non-disability pension | Disability pension, cause: | | | | |  |
| --- | --- | --- | --- | --- | --- | --- | --- |
|  | n=4204 | Mental  n=352 | Musculoskeletal  n=265 | Cardiovascular  n=71 | Nervous system  n=76 | Other diagnosis  n=217 | p |
| Birth anthropometrics, mean, SD  Weight, kg  Length, cm  Body mass index, weight/(height)^2^ | 3.34, 0.45  49.93, 1.78  13.36, 1.22 | 3.33, 0.47  49.85, 1.94  13.32, 1.27 | 3.38, 0.44  49.91, 1.51  13.51, 1.31 | 3.29, 0.48  49.62, 1.75  13.30, 1.32 | 3.44, 0.44  50.18, 1.58  13.61, 1.24 | 3.41, 0.45  50.09, 1.75  13.54, 1.16 | 0.056  0.288  0.045 |
| Birth order, %  Firstborn  Second or higher | 48.0  52.0 | 45.7  54.3 | 40.8  59.2 | 52.1  47.9 | 43.4  56.6 | 44.7  55.3 | 0.197 |
| Father’s occupational status, %  Upper middle  Lower middle  Manual worker | 20.4  25.3  54.3 | 18.1  21.6  60.3 | 6.9  21.0  72.1 | 12.1  22.1  65.8 | 12.0  20.5  67.5 | 14.1  19.3  66.6 | <0.001 |
| Highest educational attainment, %  Upper tertiary  Lower tertiary  Upper secondary  Basic or less | 9.5  24.7  23.5  42.3 | 6.5  21.9  21.6  50.0 | 2.8  19.7  26.8  50.7 | 3.9  18.5  36.8  40.8 | 3.2  13.4  24.9  58.5 | 9.5  24.7  23.5  42.3 | <0.001 |
| Adult occupational status, %  Upper middle  Lower middle  Self-employed  Manual worker | 44.6  48.1  2.6  4.7 | 31.8  56.0  2.0  10.2 | 20.8  60.0  3.4  15.8 | 28.2  56.3  2.8  12.7 | 32.9  51.3  2.6  13.2 | 23.5  57.6  6.0  12.9 | <0.001 |
| Age at transition to pension,  years, mean, SD | 61.31, 2.8 | 51.3, 8.1 | 53.0, 6.5 | 53.9, 6.4 | 51.4, 7.9 | 52.2, 7.9 | <0.001 |
